# Spillover effect of mental disorders in adolescent peer networks on likelihood of dropping out of secondary school

**DOI:** 10.1007/s00787-025-02723-8

**Published:** 2025-04-24

**Authors:** Jussi Alho, Mai Gutvilig, Ripsa Niemi, Laura Cachón-Alonso, Kaisla Komulainen, Petri Böckerman, Roger T. Webb, Marko Elovainio, Christian Hakulinen

**Affiliations:** 1 Department of Psychology, Faculty of Medicine, University of Helsinki, Helsinki, Finland; 2 School of Business and Economics, University of Jyväskylä, Jyväskylä, Finland; 3 Division of Psychology & Mental Health, University of Manchester, Manchester Academic Health Science Centre (MAHSC), Manchester, UK; 4 Finnish Institute for Health and Welfare, Helsinki, Finland; 5 Faculty of Medicine, University of Helsinki, P.O. Box 21, Haartmaninkatu 3, Helsinki, FI-00014 Finland

**Keywords:** Mental disorders, Spillover effects, Social networks, Adolescence, Educational attainment

## Abstract

Spillover effects of mental disorders on family members are well-documented, but their impact on adolescent peers remains unclear. We investigated whether having classmates with a diagnosed mental disorder in the ninth grade of lower secondary school (ages 15–16) was associated with greater likelihood of dropping out of upper secondary education (ages 16–19). We used registry data on Finnish people born between Jan 1, 1985, and Dec 31, 1997. After lower secondary school, 378,453 cohort members started general (academic) upper secondary school and 284,713 started vocational upper secondary school. Using causal mediation analysis, we disentangled the total effect of having ninth-grade classmates with a diagnosed mental disorder on upper secondary dropout into direct spillover effect and indirect effect mediated by the individual’s own mental disorder diagnosis received during upper secondary education. For academic students, having one ninth-grade classmate diagnosed with a mental disorder was associated with 6% greater likelihood of dropping out of upper secondary education (95% CI 3–9%); two diagnosed classmates, with 10% greater likelihood (6–14%); and three or more diagnosed classmates, with 16% greater likelihood (10–22%). For vocational students, the respective increases in likelihood were 5% (2–8%), 11% (6–15%), and 24% (18–32%). For both academic and vocational students, the indirect effect of dropping out mediated by one’s own mental disorder diagnosis was notably smaller than the direct effect attributable to having diagnosed ninth-grade classmates. Our results suggest that mental disorders may have spillover effects on the educational attainment of socially connected adolescent peers.

**Supplementary Information:**

The online version contains supplementary material available at 10.1007/s00787-025-02723-8.

## Introduction

Mental disorders have a substantial immediate and longer-term impact on individuals’ lives. It has been suggested that mental disorders might also socially transmit, particularly within adolescent peer networks [1]. It remains unclear, however, whether the adverse impact of mental ill-health spills over to other social domains, such as educational attainment, among peers within the same social network.

Spillover effects of mental disorders, where an individual’s mental health affects the resources and wellbeing of those in close contact, have been primarily documented within familial contexts. For instance, it is widely recognized that mental disorders can impose physical, emotional, and financial strain on caregivers and other family members [2–4]. However, research on such spillover effects among adolescent peers is lacking. Investigating these effects is crucial, as adolescence is a critical developmental phase for forming peer relationships, adopting behaviors, and making significant life decisions [5]. Moreover, investigating spillover effects among non-genetically related peers would help control for confounding factors tied to familial correlations.

Mental health and educational attainment are strongly and reciprocally linked [6]. For example, externalizing disorders, like substance use and disruptive behavior, have been shown to directly contribute to early school dropout, while internalizing disorders, such as mood and anxiety disorders, have been documented to develop also as a consequence of dropping out [7]. Moreover, lower educational attainment is associated with an increased risk of health problems, unemployment, and reduced lifetime earnings [8–10]. This, in turn, negatively impacts societal welfare through decreased tax revenues, increased reliance on public income transfers and health services, and higher engagement in socially harmful behaviors, such as crime [11]. Understanding whether early-life mental health problems influence the educational attainment of peers within the same social network provides valuable insights for developing more effective prevention and intervention strategies focused not only on individuals with mental health issues but also on those within their social network.

In the present study, we used Finnish nationwide registry data to investigate whether having classmates with a diagnosed mental disorder in the ninth grade of lower secondary school was associated with dropping out of upper secondary education. To mitigate self-selection bias (or homophily), which is common when analyzing network associations, we defined school classes as peer networks from the registry data. As earlier findings have suggested social transmission of mental disorders in adolescent peer networks [1], we used causal mediation analysis to assess how much of the total effect of having ninth-grade classmates with a mental disorder on dropout is mediated by the individual’s own diagnosis during upper secondary education. We hypothesize that mental disorders have spillover effects on the educational attainment of adolescent peers. Specifically, we expect that having classmates with a mental disorder diagnosis in lower secondary school is associated with a higher likelihood of dropping out of upper secondary school.

## Methods

### Study population

We conducted a birth cohort study of all Finnish citizens born between January 1, 1985, and December 31, 1997. Demographic, health, and school information of the cohort members were interlinked from nationwide registers based on unique identification numbers, assigned to Finnish residents since 1969. We excluded individuals who died prior to the start of or during upper secondary education or emigrated prior to the start of upper secondary education. Additionally, to enhance the stability of peer networks and improve the validity of peer network exposure, we excluded individuals who moved to the school municipality less than three years before the start of follow-up, as well as those not born in Finland who immigrated to Finland after the school-starting age (i.e., August 1 of the year they turned 7). Excluding individuals who immigrated to Finland after the school-starting age also ensures a reliable identification of those with school-age mental disorder diagnoses and those without any diagnoses.

We acquired information on school class divisions from the National Joint Application Register, which discloses ninth-grade class divisions until 2013 (i.e., those born in 1997 belong to the last birth cohort for which the information is available). Here, ninth-grade class refers to those who share a homeroom teacher and sit in the same classroom in the ninth grade. We excluded individuals with missing or insufficient class information. To exclude the smallest classes, including special education classes, and to omit incorrect registry information (e.g., implausibly large classes), we excluded individuals in classes with fewer than 10 or more than 40 students. Among the remaining (*n* = 713,687) cohort members from 861 lower secondary schools and 39,997 classes (median 6, IQR 4–7 classes per ninth grade), 47,433 had been diagnosed with a mental disorder (ICD-10 diagnoses F10–F50 or F90–F98) by the end of ninth grade and were therefore excluded from the follow-up. Of the remaining cohort members, 378,453 started general (academic) upper secondary school and 284,713 started vocational upper secondary school as their primary choice of upper secondary education after completing lower secondary school. Degree acquired by December 31, 2019 (based on information from Statistics Finland register for degrees/qualifications) was used to determine whether students graduated or dropped out of upper secondary education. For more information on the structure of the Finnish educational systems, see Supplementary Methods S1.

The Ethics Committee of the Finnish Institute for Health and Welfare approved the study plan (THL/184/6.02.01/2023§ 933). Data were linked with the permission of Statistics Finland and the Finnish Institute of Health and Welfare. Informed consent is not required for conducting register-based studies in Finland. This study followed the Strengthening the Reporting of Observational Studies in Epidemiology (STROBE) reporting guideline [12].

### Mental disorders

We obtained nationwide information on mental disorders from healthcare registers of the Finnish Institute for Health and Welfare, which contain information on all inpatient hospital admissions in Finland since 1970, hospital outpatient care since 1998, and primary care since 2011. This means that, for the first birth cohort included in the study (i.e., those born in 1985), mental disorder diagnoses were reliably recorded from age 13 onward—coinciding with the start of lower secondary school—thereby allowing sufficient time for peer network exposure. Mental disorder diagnoses were based on the International Statistical Classification of Diseases spectrum Health Problems, Eight Revision (ICD-8) from 1970 to 1986, Ninth Revision (ICD-9) from 1987 to 1995, and Tenth Revision (ICD-10) since 1996. Some primary care facilities also use the International Classification of Primary Care, Second Edition (ICPC-2) alongside the ICD-10.

We used the following mental disorder diagnosis categories for the study population: substance use disorders (F10–F19), schizophrenia spectrum disorders (F20–F29), mood disorders (F30–F39), anxiety disorders (F40–F48), eating disorders (F50), and behavioral and emotional disorders (F90–F98). Additionally, we constructed categories of internalizing disorders (F30–F39, F40–F48, F93–F94; comprising mood, anxiety, emotional, and social functioning disorders) and externalizing disorders (F10–F19, F90–F92; comprising substance use, hyperkinetic, and conduct disorders).

### Covariates

We included the following variables as covariates (see Table 1 and Supplementary Table S1): sex; birth year; degree of urbanicity in residential location based on the Finnish Environment Institute’s urban-rural classification; morbidity index in municipality by the Finnish Institute for Health and Welfare in quintiles (as data were not available for 2001, data from 2002 were used instead); proportion of residents without upper secondary or higher education in the municipality; proportion of unemployed residents in the municipality, number of students in the same ninth-grade class (i.e., classmates); total number of students in the entire ninth grade; achievement in lower secondary school as measured by the final grade average; parental education level at the time of child’s ninth grade; parental income level relative to study population at the time of child’s ninth grade in quintiles; and parental mental health disorder history at the time of child’s ninth grade. The median population of Finnish municipalities in 2001–2013 was 6530 inhabitants. The school achievement measure was obtained from the National Joint Application Register and calculated based on all compulsory and optional subjects.

**Table 1:** Descriptive statistics of the study population

	Mental disorder diagnosed before follow-up		Follow-up population			
			Academic students		Vocational students	
	n	%	n	%	n	%
**Sex**						
Male	22 147	46.7	164 003	43.3	171 919	60.4
Female	25 286	53.3	214 450	56.7	112 794	39.6
**Birth year**						
1985	1936	4.1	31 809	8.4	21 546	7.6
1986	2092	4.4	31 032	8.2	20 805	7.3
1987	2243	4.7	30 498	8.1	20 390	7.2
1988	2700	5.7	31 251	8.3	21 982	7.7
1989	2843	6.0	30 511	8.1	22 457	7.9
1990	3330	7.0	30 105	8.0	23 901	8.4
1991	3544	7.5	29 218	7.7	23 662	8.3
1992	3882	8.2	28 590	7.6	23 813	8.4
1993	4384	9.2	28 241	7.5	23 312	8.2
1994	4671	9.8	27 975	7.4	23 287	8.2
1995	5013	10.6	27 516	7.3	22 151	7.8
1996	5354	11.3	26 379	7.0	20 010	7.0
1997	5441	11.5	25 328	6.7	17 397	6.1
Mental disorder diagnosis*						
F10-F19 Substance misuse disorders	3458	7.3	1 826	0.5	7 247	1.9
F20-F29 Schizophrenia spectrum disorders	1142	2.4	983	0.3	1 464	0.4
F30-F39 Mood disorders	12 472	26.3	8 360	2.2	12 939	3.4
F40-F48 Anxiety disorders	13 730	28.9	9 212	2.4	16 144	4.3
F50 Eating disorders	4012	8.5	2 359	0.6	953	0.3
F90-F98 Behavioral and emotional disorders	26 532	55.9	2 404	0.6	3 512	0.9
Internalizing disorders	26 303	55.5	14 724	3.9	22 624	6.0
Externalizing disorders	14 152	29.8	2 200	0.6	8 171	2.2
Any of the above	47 433	100.0	17 878	4.7	27 922	7.4
**Graduated from upper secondary education**						
Yes	35 626	75.1	343 702	90.8	253 024	88.9
No	11 109	23.4	34 751	9.2	31 689	11.1

* For the follow-up population, mental disorder diagnosis received during upper secondary education

### Statistical analysis

Before utilizing mediation analysis, we examined associations between the exposure, mediator, and outcome variables. Specifically, we used logistic regression to separately estimate whether those who had ninth-grade classmates with a mental disorder diagnosis were more likely to drop out or receive a mental disorder diagnosis during upper secondary education, and whether those who received a mental disorder diagnosis during upper secondary education were more likely to dropout.

**Fig. 1:**
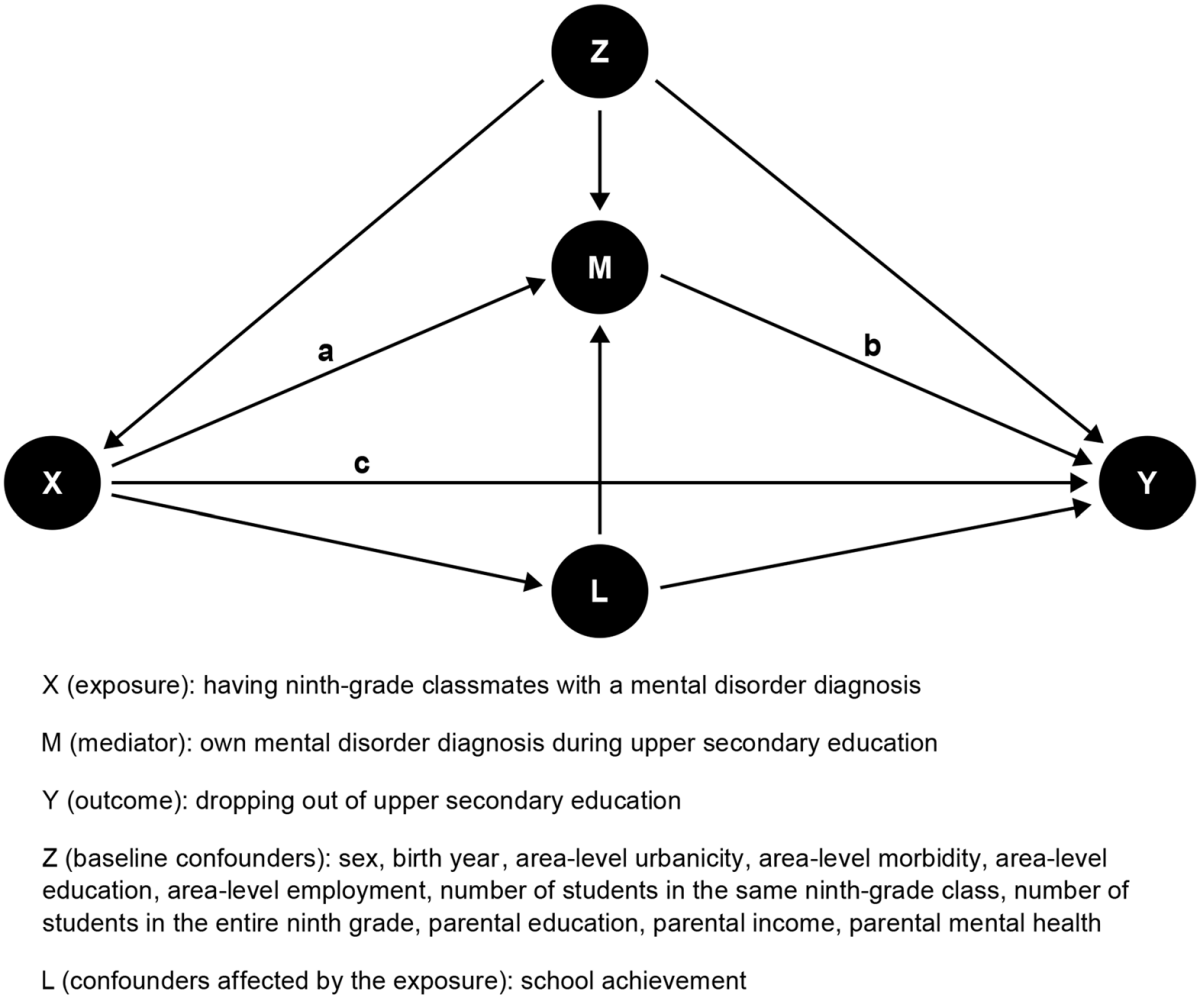
Directed acyclic graph of the association between having ninth-grade classmates with a mental disorder diagnosis and dropping out of upper secondary education. The indirect effect operates via path a + b and the direct effect operates via path c. The total effect is the sum of paths a + b and c

Second, we used causal mediation analysis [13] to disentangle the total effect of having ninth-grade classmates with a mental disorder on dropping out of upper secondary education into a direct spillover effect and an indirect effect mediated through the possible transmission of mental disorders, with the individual’s own diagnosis received during upper secondary education as a mediator (Fig. 1). In this framework, we used the marginal structural model approach [14], with logistic regression models for the exposure, mediator, and outcome. The randomized analogues for the total natural direct and indirect effects, as well as the exposure-mediator interaction effects (both reference and mediated interaction), were estimated using direct counterfactual imputation with bootstrapped standard errors using 200 samples [15]. Reference interaction is the direct combined effect of exposure and mediator on the outcome, independent of the mediation pathway, while mediated interaction is the combined effect of exposure and mediator on the outcome through the indirect pathway. In the primary analyses, we used a four-level exposure variable for the number of diagnosed ninth-grade classmates (none, one, two, or three or more), binary mediator variable for mental disorder diagnosis received during upper secondary education, and binary outcome variable for dropping out of upper secondary education.

As a sensitivity analysis, we estimated the models separately for each mental disorder diagnosis category, using the same category for both the exposure and mediator. Since the number of cases with multiple diagnosed classmates was low for some diagnosis categories, we used a binary variable indicating the presence or lack of diagnosed ninth-grade classmates as an exposure in the diagnosis-specific analyses. As an additional sensitivity analysis, we limited the time window for one’s own mental disorder diagnosis to the first year of upper secondary education to account for variability in upper secondary education duration between completers (academic students: mean 3.1 [SD 0.5] years; vocational students: mean 3.6 [SD 1.9] years) and dropouts (academic students: mean 2.6 [SD 1.6] years; vocational students: mean 4.3 [SD 2.4] years). The statistical analyses were done using Stata (v16.1) and R Statistical Software (v4.2.2; R Core Team 2021) using CMAverse (v0.1.0) [16] and lme4 (v1.1.31) [17] packages.

## Results

Descriptive statistics of the study population are shown in Table 1 (for additional descriptive statistics, see Supplementary Table S1). In the follow-up population, 34,751 (9.2%) of those who started general upper secondary school and 31,689 (11.1%) of those who started vocational upper secondary school dropped out, respectively. Among academic students, 122,894 (32.5%) had one ninth-grade classmate with a diagnosed mental disorder, 66,630 (17.6%) had two, and 45,712 (12.1%) had three or more. Among vocational students, 91,490 (32.1%) had one diagnosed ninth-grade classmate, 49,836 (17.5%) had two, and 35,774 (12.6%) had three or more.

Results from the logistic regression analysis (Table 2) showed that, for academic students, one ninth-grade classmate with a mental disorder diagnosis was associated with 8% greater likelihood of being diagnosed with a mental disorder during upper secondary education (OR = 1.08, 95% CI 1.04–1.12), two diagnosed ninth-grade classmates was associated with 14% greater likelihood (OR = 1.14, 95% CI 1.09–1.19), and three or more diagnosed ninth-grade classmates was associated with 23% greater likelihood (OR = 1.23, 95% CI 1.17–1.29). For vocational students, one diagnosed ninth-grade classmate was associated with 4% greater likelihood (OR = 1.04, 95% CI 1.01–1.07), two diagnosed ninth-grade classmates was associated with 11% greater likelihood (OR = 1.11, 95% CI 1.07–1.15), and three or more diagnosed ninth-grade classmates was associated with 17% greater likelihood of a mental disorder diagnosis during upper secondary education (OR = 1.17, 95% CI 1.12–1.22).

**Table 2:** Associations between having ninth-grade classmates with a mental disorder diagnosis and receiving a mental disorder diagnosis during upper secondary education (a), receiving a mental disorder diagnosis during upper secondary education and dropping out of upper secondary education (b), and having ninth-grade classmates with a mental disorder diagnosis and dropping out of upper secondary education (c). Sections a, b, and c correspond to paths a, b, and c in Fig. 1, respectively

a	Ninth-grade classmates with a mental disorder diagnosis						
	None (ref.)	One		Two		Three or more	
	*n*	*n*	OR (95% CI)	*n*	OR (95% CI)	*n*	OR (95% CI)
**Mental disorder diagnosed during upper secondary education**							
Academic students	5401	5720	1.08 (1.04 to 1.12)	3635	1.14 (1.09 to 1.19)	3122	1.23 (1.17 to 1.29)
Vocational students	9025	8787	1.04 (1.01 to 1.07)	5492	1.11 (1.07 to 1.15)	4618	1.17 (1.12 to 1.22)

b	Mental disorder diagnosed during upper secondary education						
	No (ref.)	**Yes**					
	*n*	*n*	OR (95% CI)				
**Dropped out of upper secondary education**							
Academic students	31 001	3750	2.76 (2.65 to 2.88)				
Vocational students	24 300	7389	2.87 (2.78 to 2.97)				

c	Ninth-grade classmates with a mental disorder diagnosis						
	None (ref.)	One		Two		Three or more	
	*n*	*n*	OR (95% CI)	*n*	OR (95% CI)	*n*	OR (95% CI)
**Dropped out of upper secondary education**							
Academic students	13 021	11,252	1.06 (1.03 to 1.09)	6171	1.10 (1.06 to 1.14)	4307	1.15 (1.10 to 1.20)
Vocational students	11 225	10,036	1.04 (1.01 to 1.08)	5818	1.10 (1.06 to 1.14)	4610	1.17 (1.12 to 1.22)

The logistic regression models were adjusted for sex, birth year, area-level urbanicity, area-level morbidity, area-level education, area-level employment, number of students in the same ninth-grade class, total number of students in the entire ninth grade, parental education, parental income, parental mental health, and school achievement. For a sensitivity analysis with a restricted time window for mental disorder diagnosis during upper secondary education, see Supplementary Table S3

Receiving a mental disorder diagnosis during upper secondary education was associated with greater likelihood of dropping out for both academic (OR = 2.76, 95% CI 2.65–2.88) and vocational students (OR = 2.87, 95% CI 2.78–2.97). For academic students, one ninth-grade classmate with a mental disorder diagnosis was associated with 6% greater likelihood of dropping out of upper secondary education (OR = 1.06, 95% CI 1.03–1.09), two diagnosed ninth-grade classmates was associated with 10% greater likelihood (OR = 1.10, 95% CI 1.06–1.14), and three or more diagnosed ninth-grade classmates was associated with 15% greater likelihood (OR = 1.15, 95% CI 1.10–1.20). Similarly, for vocational students, one diagnosed ninth-grade classmate was associated with 4% (OR = 1.04, 95% CI 1.01–1.08), two diagnosed ninth-grade classmates was associated with 10% (OR = 1.10, 95% CI 1.06–1.14), and three or more diagnosed ninth-grade classmates was associated with 17% greater likelihood (OR = 1.17, 95% CI 1.12–1.22) of dropping out of upper secondary education. For diagnosis-specific results, see Supplementary Table S2.

Results from the causal mediation analysis (Table 3) showed that, for academic students, one ninth-grade classmate with a mental disorder diagnosis was associated with 6% greater likelihood of dropping out of upper secondary education (OR = 1.06, 95% CI 1.03–1.09), two diagnosed ninth-grade classmates was associated with 10% greater likelihood (OR = 1.10, 95% CI 1.06–1.14), and three or more diagnosed ninth-grade classmates was associated with 16% greater likelihood (OR = 1.16, 95% CI 1.10–1.22). For vocational students, causal mediation analysis showed that one diagnosed ninth-grade classmate was associated with 5% greater likelihood (OR = 1.05, 95% CI 1.02–1.08), two diagnosed ninth-grade classmates was associated with 11% greater likelihood (OR = 1.11, 95% CI 1.06–1.15), and three or more diagnosed ninth-grade classmates was associated with 24% greater likelihood (OR = 1.24, 95% CI 1.18–1.32) of dropping out of upper secondary education. For both the academic and vocational students, the indirect effects of dropping out attributable to one’s own mental disorder diagnosis received during upper secondary education were small, with the overall proportion mediated ranging from 8 to 12%. The reference interaction was notable only for academic and vocational students who had three or more diagnosed ninth-grade classmates, with the overall proportion attributable to interaction at 15%. The mediated interaction did not show relevance for any of the associations.

**Table 3:** Effect of having one or more than one ninth-grade classmate with a mental disorder diagnosis on dropping out of upper secondary education, with one’s own mental disorder diagnosis received during upper secondary education as a mediator

	Ninth-grade classmates with a diagnosed mental disorder		
	One	Two	Three or more
**Academic students**			
Total effect	1.06 (1.03 to 1.09)	1.10 (1.06 to 1.14)	1.16 (1.10 to 1.22)
Direct effect	1.06 (1.02 to 1.08)	1.09 (1.05 to 1.13)	1.14 (1.08 to 1.20)
Indirect effect	1.01 (1.00 to 1.01)	1.01 (1.00 to 1.01)	1.02 (1.01 to 1.02)
Exposure-mediator interaction	1.00 (0.99 to 1.01)	1.01 (1.00 to 1.03)	1.02 (1.00 to 1.04)
**Vocational students**			
Total effect	1.05 (1.02 to 1.08)	1.11 (1.06 to 1.15)	1.24 (1.18 to 1.32)
Direct effect	1.04 (1.01 to 1.07)	1.09 (1.05 to 1.14)	1.21 (1.15 to 1.29)
Indirect effect	1.00 (1.00 to 1.01)	1.01 (1.01 to 1.02)	1.02 (1.02 to 1.03)
Exposure-mediator interaction	1.00 (0.98 to 1.01)	1.00 (0.98 to 1.02)	1.03 (1.01 to 1.07)

Odds ratios with 95% CIs for the total effect, (total natural) direct effect, (total natural) indirect effect, and exposure-mediator (reference) interaction shown separately for academic and vocational students. The causal mediation analysis models included the following baseline confounders: sex, birth year, area-level urbanicity, area-level morbidity, area-level education, area-level employment, number of students in the same ninth-grade class, total number of students in the entire ninth grade, parental education, parental income, and parental mental health. School achievement was included as a confounder affected by the exposure. For a sensitivity analysis with a restricted time window for mental disorder diagnosis during upper secondary education, see Supplementary Table S4

Diagnosis-specific causal mediation analysis (Table 4) revealed that having ninth-grade classmates with a diagnosed substance use, mood, anxiety, or behavioral and emotional disorder was associated with greater likelihood of dropping out for academic students. Similar associations were found for the broader internalizing or externalizing disorder diagnosis categories. Among these diagnosis categories, a notable indirect effect on dropping out was due to the student’s own mood or anxiety (or internalizing) disorder diagnosis received during upper secondary education. The reference interaction was notable for mood and behavioral and emotional disorders as well as for the internalizing and externalizing disorder categories. For vocational students, the likelihood of dropping out was greater if their ninth-grade classmates had a diagnosis of substance use, schizophrenia spectrum, mood, anxiety, or behavioral and emotional disorders. The indirect effect mediated by the vocational student’s own diagnosis during upper secondary education was notable for substance use, mood, anxiety, and behavioral and emotional disorders. Similarly to academic students, the total and direct effect of dropping out for vocational students was notable if the ninth-grade classmates had internalizing or externalizing disorder diagnoses; however, unlike for academic students, the reference interaction was notable only for externalizing disorders, and the indirect effect mediated by the student’s own diagnosis was notable for both internalizing and externalizing disorders. The results remained similar when restricting the time window for the student’s own mental disorder diagnosis to the first year of upper secondary education (Supplementary Tables S3–S6).

**Table 4:** Effect of ninth-grade classmates with a specific mental disorder diagnosis on dropping out of upper secondary education, with own mental disorder diagnosis received during upper secondary education as a mediator

	Total effect		Direct effect		Indirect effect		Exposure-mediator interaction	
	OR	95% CI	OR	95% CI	OR	95% CI	OR	95% CI
**Academic students**								
F10–F19 Substance use disorders	1.076	1.029, 1.125	1.075	1.029, 1.125	1.001	0.999, 1.002	1.006	1.000, 1.013
F20–F29 Schizophrenia spectrum disorders	0.986	0.902, 1.131	0.987	0.903, 1.133	1.000	0.997, 1.002	0.994	0.985, 1.007
F30–F39 Mood disorders	1.069	1.043, 1.095	1.059	1.032, 1.085	1.010	1.007, 1.013	1.011	1.005, 1.019
F40–F48 Anxiety disorders	1.048	1.023, 1.080	1.045	1.020, 1.077	1.003	1.001, 1.004	1.001	0.994, 1.009
F50 Eating disorders	0.994	0.946, 1.038	0.994	0.945, 1.038	1.001	0.999, 1.002	1.001	0.989, 1.015
F90–F98 Behavioral and emotional disorders	1.075	1.049, 1.098	1.074	1.049, 1.097	1.001	1.000, 1.003	1.008	1.003, 1.012
Internalizing disorders	1.079	1.052, 1.107	1.071	1.044, 1.099	1.008	1.006, 1.011	1.011	1.003, 1.019
Externalizing disorders	1.092	1.065, 1.123	1.091	1.064, 1.121	1.001	1.000, 1.002	1.009	1.004, 1.014
Any of the above	1.074	1.052, 1.103	1.067	1.044, 1.093	1.006	1.005, 1.010	1.005	0.997, 1.014
**Vocational students**								
F10–F19 Substance use disorders	1.089	1.043, 1.139	1.084	1.038, 1.131	1.006	1.003, 1.012	1.021	1.008, 1.034
F20–F29 Schizophrenia spectrum disorders	1.122	1.044, 1.198	1.121	1.043, 1.194	1.000	0.999, 1.003	1.007	0.995, 1.022
F30–F39 Mood disorders	1.072	1.040, 1.103	1.064	1.034, 1.095	1.006	1.002, 1.010	1.006	0.995, 1.016
F40–F48 Anxiety disorders	1.085	1.053, 1.114	1.080	1.048, 1.108	1.004	1.001, 1.008	1.017	1.006, 1.029
F50 Eating disorders	1.030	0.990, 1.075	1.030	0.990, 1.074	1.001	0.999, 1.002	1.005	0.999, 1.014
F90–F98 Behavioral and emotional disorders	1.087	1.059, 1.114	1.085	1.057, 1.112	1.001	1.000, 1.004	1.009	1.004, 1.015
Internalizing disorders	1.083	1.055, 1.108	1.073	1.048, 1.098	1.009	1.005, 1.012	1.004	0.991, 1.014
Externalizing disorders	1.073	1.048, 1.102	1.069	1.043, 1.097	1.004	1.002, 1.008	1.012	1.003, 1.020
Any of the above	1.092	1.067, 1.115	1.081	1.054, 1.108	1.009	1.005, 1.013	1.004	0.993, 1.018

Odds ratios with 95% CIs for the total effect, (total natural) direct effect, (total natural) indirect effect, and exposure-mediator (reference) interaction shown separately for academic and vocational upper secondary students. The causal mediation analysis models included the following baseline confounders: sex, birth year, area-level urbanicity, area-level morbidity, area-level education, area-level employment, number of students in the same ninth-grade class, total number of students in the entire ninth grade, parental education, parental income, and parental mental health. School achievement was included as a confounder affected by the exposure. For a sensitivity analysis with a restricted time window for mental disorder diagnosis during upper secondary education, see Supplementary Table S6

## Discussion

In our analysis of nationwide registry data, we found that having classmates with a diagnosed mental disorder in the ninth grade of lower secondary school (ages 15–16) was associated with a greater likelihood of dropping out of upper secondary education (ages 16–19). While having ninth-grade classmates with a diagnosed mental disorder was associated with greater likelihood of being diagnosed with a mental disorder during upper secondary education, and being diagnosed during upper secondary education was associated with greater likelihood of dropping out, the indirect effect of dropping out attributable to one’s own mental disorder diagnosis was notably smaller than the direct effect of having ninth-grade classmates with a mental disorder diagnosis. Additionally, the direct effect showed a dose-response relationship, with a higher likelihood of dropping out as the number of diagnosed ninth-grade classmates increased. These associations were observed after adjusting for a broad array of individual-, parental-, school-, and area-level confounders.

Previous empirical studies on health-related social network influences and spillover effects have shown the interdependence of health among socially connected individuals [18, 19]. Among adolescents, social network effects are mostly documented as peer influences on substance use disorders and related health-risk behaviors, such as smoking and alcohol use [20–23]. Generally, peer effects appear to significantly impact social and health behavior outcomes, particularly during adolescence [5, 24, 25]. Spillover effects, on the other hand, have mainly been documented in the context of illness in family influencing various social and health outcomes among family members, both within and across generations [26]. To our knowledge, the present study is the first to use nationwide register data to investigate whether the potential spillover effects of mental disorders may extend to a social outcome, specifically educational dropout, among adolescent peers.

Corroborating previous findings suggesting social transmission of mental disorders [1, 27, 28], we observed greater likelihood of receiving a mental disorder diagnosis during upper secondary education for those who had ninth-grade classmates with a mental disorder diagnosis. This association was clearer for academic than for vocational students. We also observed that students who were diagnosed with a mental disorder during upper secondary education were more likely to drop out, which is consistent with earlier findings identifying psychiatric problems in adolescence as a strong predictor of low educational attainment [7, 29–31]. Our novel investigation has revealed direct relationship between having peers with a mental disorder diagnosis in the ninth grade of lower secondary school and a greater likelihood of dropping out of upper secondary education. This suggests that the social reach of mental disorders may extend beyond caregivers and family members to peers within the same social network.

The observed association between having ninth-grade classmates with externalizing disorders and dropping out of upper secondary education was predominantly direct. In contrast, the association between having ninth-grade classmates with internalizing disorders and dropping out was, to a larger extent, mediated by the student’s own mental disorder diagnosis. This aligns with earlier findings suggesting that mood and anxiety disorders are among the most socially transmissible mental disorders among adolescents [1]. We also observed a notable reference interaction between ninth-grade classmates with externalizing disorders and a student’s own externalizing disorder diagnosed during upper secondary education. This may indicate that students with emerging externalizing tendencies, such as excessive substance use, might naturally gravitate toward peers with similar behaviors, reinforcing shared disengagement from school rather than direct transmission of externalizing symptoms from classmates to otherwise healthy peers. This interpretation aligns with well-established findings on peer selection processes among adolescents, particularly for externalizing behaviors (e.g., substance use, delinquency), where students often seek out similar peers [32, 33]. The predominantly direct effect of having ninth-grade classmates with an externalizing disorder on dropping out also extends earlier findings showing that an individual’s own externalizing disorder, rather than internalizing disorder, directly contributes to the likelihood of school dropout [7]. This spillover effect might reflect broader classroom disruptions (e.g., changes in peer norms or teacher attention) that increase dropout risk for all students.

While the effect of having ninth-grade classmates diagnosed with a mental disorder on dropping out of upper secondary education was largely similar for both academic and vocational students, having ninth-grade classmates with diagnosed schizophrenia spectrum disorders was associated with a greater likelihood of dropping out among vocational, but not academic, students. Also, vocational students were more likely to be diagnosed with an externalizing disorder during upper secondary education if they had peers with an externalizing disorder either in their ninth-grade class or whole ninth grade. In contrast, academic students with peers who had an internalizing disorder either in the same ninth-grade class or whole ninth grade were more likely to be diagnosed with an internalizing disorder.

### Strengths and limitations

The present study’s main strengths include its use of a nationwide study population, incorporation of linked primary and secondary health care registry data, and leveraging institutionally imposed peer networks to mitigate self-selection bias. Nevertheless, our findings need to be interpreted considering the study’s limitations. First, residual confounding due to unmeasured or inaccurately measured covariates (e.g., from socially deprived neighborhoods) cannot be ruled out. Second, some individuals with underlying mental disorders may not seek healthcare services, implying that reported diagnoses likely underestimate true disorder prevalence. Relatedly, since primary care data became available in the healthcare registers only from 2011 onwards, the underestimation of mental disorder prevalence is more pronounced in earlier birth cohorts. The observed increasing trend in diagnosed mental disorders across birth cohorts likely reflects both true rises in adolescent mental health issues [34] and improved case detection due to greater access to mental health services and more comprehensive data availability, which may influence our estimates. Third, school class is a relatively crude proxy for a peer network and registry information on class divisions were available only for the ninth grade. That being said, classes within the Finnish educational system, especially in lower secondary education (grades 7–9), tend to be stable with infrequent transitions between them [35]. Finally, given Finland’s small and relatively homogeneous population as well as universal healthcare covering all citizens, studies in other high-income countries are needed to confirm the generalizability of our findings.

## Conclusions

Based on a nationwide study population of over 700 000 individuals, the findings of this cohort study suggest that having classmates with a mental disorder diagnosis in the ninth grade of lower secondary school was associated with a greater likelihood of dropping out of upper secondary education. Moreover, our findings showed that having ninth-grade classmates with a diagnosed mental disorder was associated with greater likelihood of receiving a mental disorder diagnosis during upper secondary education and, in turn, receiving a mental disorder diagnosis during upper secondary education was associated with greater likelihood of dropping out. However, causal mediation analysis showed that the indirect effect of dropping out mediated by one’s own mental disorder diagnosis was notably smaller than the direct effect attributable to having ninth-grade classmates with a diagnosed mental disorder. This implies that, in addition to the previously suggested social transmission of mental disorders among adolescent peers [1], mental disorders may have spillover effects that extend to socially connected adolescent peers. Suggesting that adolescents’ mental health may affect not only their own outcomes but also have broader impacts on their peers’ educational trajectories, our findings underscore the need for early interventions in healthcare and schools. Policies should focus on increasing access to school-based mental health services, implementing peer support programs, and promoting early identification strategies. Future research should elucidate the mechanisms underlying these spillover associations.

## Electronic supplementary material

Below is the link to the electronic supplementary material.

Supplementary Material 1

## Data Availability

No datasets were generated or analysed during the current study.
