# Supplementary material for: Spillover effect of mental disorders in adolescent peer networks on likelihood of dropping out of secondary school
Source: Eur Child Adolesc Psychiatry. 2025 Apr 24;34(10):3105–14. doi: 10.1007/s00787-025-02723-8 (PMC12592305; doi:10.1007/s00787-025-02723-8)
Supplement: Supplementary file 1 — Supplementary Material 1 [file 787_2025_2723_MOESM1_ESM.docx]

**Supplementary Material**

**CONTENTS**

[Methods S1: Structure of the Finnish comprehensive and upper secondary educational systems 2](#_Toc182820367)

[Table S1: Additional descriptive statistics of the study population. 3](#_Toc182820368)

[Table S2: Diagnosis-specific results from the logistic regression analysis. 4](#_Toc182820369)

[Table S3: Results from the logistic regression analysis with a restricted time window for mental disorder diagnosis during upper secondary education. 5](#_Toc182820370)

[Table S4: Results from the causal mediation analysis with a restricted time window for mental disorder diagnosis during upper secondary education. 6](#_Toc182820371)

[Table S5: Diagnosis-specific results from the logistic regression analysis with a restricted time window for mental disorder diagnosis during upper secondary education. 7](#_Toc182820372)

[Table S6: Diagnosis-specific results from the causal mediation analysis with a restricted time window for mental disorder diagnosis during upper secondary education 8](#_Toc182820373)

[References 9](#_Toc182820374)

## Methods S1: Structure of the Finnish comprehensive and upper secondary educational systems

In Finland, the nine-year comprehensive education starts the year children turn seven and continues until they are around 16 years old. The comprehensive school system includes primary (grades 1–6) and lower secondary (grades 7–9) schools, and is uniform in nature (i.e., does not include different tracks for academic or vocational education). Comprehensive school students typically attend the municipal school closest to their home, with fewer than 2% of students opting for a private or publicly-funded school beyond their municipality.^1^

After the comprehensive education, students can enter upper secondary education, which requires a comprehensive school certificate. Applications for upper secondary education are processed through a joint application system, with selections based on students’ school reports. The upper secondary level includes general (academic) and vocational education. General upper secondary education (equivalent to high school) is structured around courses instead of traditional year classes and culminates in a nationally comparable matriculation examination. Vocational education is structured in year classes. Both general and vocational education usually take three years to complete. Comprehensive and upper secondary education in Finland are free of charge, without any tuition fees. However, in upper secondary education, students are required to cover the costs of their study materials.

## Table S1: Additional descriptive statistics of the study population.

|  |  | **Follow-up population** | |
| --- | --- | --- | --- |
|  | **Mental disorder diagnosed before follow-up,  n (%) or mean (SD)** | **Academic students,  n (%) or mean (SD)** | **Vocational students,  n (%) or mean (SD)** |
| **Diagnosed ninth-grade classmates** |  |  |  |
| None | na | 143 217 (37.8) | 107 613 (37.8) |
| One | na | 122 894 (32.5) | 91 490 (32.1) |
| More than one | na | 112 342 (29.7) | 85 610 (30.1) |
| **Number of students in the same ninth-grade class** | 19.5 (3.9) | 20 (3.6) | 19 (3.5) |
| **Total number of students in the entire ninth grade** | 109.9 (46.2) | 113 (44.6) | 110 (46.0) |
| **Lower secondary school achievement** |  |  |  |
| Unknown | 1662 (3.5) | 1 484 (0.4) | 2 459 (0.9) |
| Quintile 1 (lowest) | 16 007 (33.7) | 6 818 (1.8) | 117 055 (41.1) |
| Quintile 2 | 10 387 (21.9) | 39 368 (10.4) | 91 392 (32.1) |
| Quintile 3 | 7771 (16.4) | 87 430 (23.1) | 46 673 (16.4) |
| Quintile 4 | 6096 (12.9) | 114 267 (30.2) | 20 391 (7.2) |
| Quintile 5 (highest) | 5510 (11.6) | 129 086 (34.1) | 6 743 (2.4) |
| **Area-level urbanicity** |  |  |  |
| Unknown | 1775 (3.7) | 1 297 (0.3) | 2 018 (0.7) |
| Urban | 27 629 (58.3) | 94 148 (24.9) | 92 404 (32.5) |
| Semi-urban | 7750 (16.3) | 68 068 (18.0) | 56 948 (20.0) |
| Rural | 10 279 (21.7) | 214 940 (56.8) | 133 343 (46.8) |
| **Area-level morbidity** |  |  |  |
| Quintile 1 (lowest) | 13 038 (27.5) | 86 617 (22.9) | 43 316 (15.2) |
| Quintile 2 | 9511 (20.1) | 76 735 (20.3) | 55 081 (19.3) |
| Quintile 3 | 9085 (19.1) | 70 946 (18.7) | 61 964 (21.8) |
| Quintile 4 | 7982 (16.8) | 73 774 (19.5) | 60 851 (21.4) |
| Quintile 5 (highest) | 7817 (16.5) | 70 381 (18.6) | 63 501 (22.3) |
| **Area-level employment** |  |  |  |
| Quintile 1 (highest) | 10 919 (23.0) | 84 312 (22.3) | 52 032 (18.3) |
| Quintile 2 | 10 162 (21.4) | 74 503 (19.7) | 52 169 (18.3) |
| Quintile 3 | 9412 (19.8) | 72 024 (19.0) | 58 214 (20.4) |
| Quintile 4 | 8710 (18.4) | 70 860 (18.7) | 59 851 (21.0) |
| Quintile 5 (lowest) | 8230 (17.4) | 76 754 (20.3) | 62 447 (21.9) |
| **Area-level education** |  |  |  |
| Quintile 1 (highest) | 11 923 (25.1) | 77 939 (20.6) | 41 039 (14.4) |
| Quintile 2 | 10 491 (22.1) | 79 334 (21.0) | 50 743 (17.8) |
| Quintile 3 | 10 990 (23.2) | 77 329 (20.4) | 65 994 (23.2) |
| Quintile 4 | 8164 (17.2) | 74 131 (19.6) | 63 911 (22.4) |
| Quintile 5 (lowest) | 5865 (12.4) | 69 720 (18.4) | 63 026 (22.1) |
| **Mother's education** |  |  |  |
| Primary | 8387 (17.7) | 33 977 (9.0) | 54 311 (19.1) |
| Secondary | 20 663 (43.6) | 132 617 (35.0) | 152 135 (53.4) |
| High | 18 383 (38.8) | 211 859 (56.0) | 78 267 (27.5) |
| **Father's education** |  |  |  |
| Primary | 13 594 (28.7) | 64 115 (16.9) | 86 338 (30.3) |
| Secondary | 20 252 (42.7) | 140 145 (37.0) | 147 396 (51.8) |
| High | 13 587 (28.6) | 174 193 (46.0) | 50 979 (17.9) |
| **Mother's income level** |  |  |  |
| Unknown | 1222 (2.6) | 3 852 (1.0) | 3 970 (1.4) |
| Quintile 1 (lowest) | 11340 (23.9) | 58 792 (15.5) | 69 500 (24.4) |
| Quintile 2 | 8146 (17.2) | 65 108 (17.2) | 66 988 (23.5) |
| Quintile 3 | 8225 (17.3) | 72 352 (19.1) | 59 874 (21.0) |
| Quintile 4 | 9207 (19.4) | 79 616 (21.0) | 51 737 (18.2) |
| Quintile 5 (highest) | 9293 (19.6) | 98 733 (26.1) | 32 644 (11.5) |
| **Father's income level** |  |  |  |
| Unknown | 4200 (8.9) | 14 197 (3.8) | 15 433 (5.4) |
| Quintile 1 (lowest) | 10 710 (22.6) | 57 817 (15.3) | 66 288 (23.3) |
| Quintile 2 | 7886 (16.6) | 62 987 (16.6) | 64 422 (22.6) |
| Quintile 3 | 8102 (17.1) | 68 249 (18.0) | 59 109 (20.8) |
| Quintile 4 | 8302 (17.5) | 78 389 (20.7) | 48 859 (17.2) |
| Quintile 5 (highest) | 8233 (17.4) | 96 814 (25.6) | 30 602 (10.7) |
| **Mother's mental disorder** |  |  |  |
| No | 36 488 (76.9) | 348 547 (92.1) | 253 638 (89.1) |
| Yes | 10 945 (23.1) | 29 906 (7.9) | 31 075 (10.9) |
| **Father's mental disorder** |  |  |  |
| No | 38 381 (80.9) | 347 852 (91.9) | 249 233 (87.5) |
| Yes | 9052 (19.1) | 30 601 (8.1) | 35 480 (12.5) |

Table S2: Diagnosis-specific results from the logistic regression analysis. Associations between (A) having ninth-grade classmates with a mental disorder diagnosis and receiving a mental disorder diagnosis during upper secondary education, (B) receiving a mental disorder diagnosis during upper secondary education and dropping out of upper secondary education, and (C) having ninth-grade classmates with a mental disorder diagnosis and dropping out of upper secondary education (right).

|  | **A** |  |  | **B** |  |  | **C** |  |
| --- | --- | --- | --- | --- | --- | --- | --- | --- |
|  | n | OR (95% CI) |  | n | OR (95% CI) |  | n | OR (95% CI) |
| **Academic students** |  |  |  |  |  |  |  |  |
| F10–F19 Substance use disorders | 156 | 1.05 (0.89 to 1.24) |  | 542 | 3.39 (3.03 to 3.79) |  | 2624 | 1.04 (1.00 to 1.09) |
| F20–F29 Schizophr. spectr. disorders | 23 | 0.88 (0.58 to 1.33) |  | 420 | 7.51 (6.51 to 8.67) |  | 885 | 0.99 (0.92 to 1.07) |
| F30–F39 Mood disorders | 2510 | 1.21 (1.15 to 1.27) |  | 2225 | 3.68 (3.48 to 3.89) |  | 8462 | 1.07 (1.04 to 1.10) |
| F40–F48 Anxiety disorders | 3073 | 1.07 (1.02 to 1.12) |  | 1846 | 2.44 (2.30 to 2.58) |  | 9028 | 1.05 (1.02 to 1.08) |
| F50 Eating disorders | 273 | 1.04 (0.92 to 1.19) |  | 287 | 1.92 (1.67 to 2.19) |  | 3212 | 1.00 (0.96 to 1.04) |
| F90–F98 Behav. and emot. disorders | 1200 | 1.05 (0.97 to 1.14) |  | 633 | 3.34 (3.02 to 3.70) |  | 14 630 | 1.08 (1.05 to 1.10) |
| Internalising disorders | 7485 | 1.14 (1.10 to 1.18) |  | 3236 | 2.87 (2.75 to 3.01) |  | 15 075 | 1.08 (1.05 to 1.11) |
| Externalising disorders | 630 | 1.03 (0.94 to 1.13) |  | 681 | 3.57 (3.22 to 3.95) |  | 8872 | 1.08 (1.06 to 1.11) |
| Any of the above | 12 477 | 1.12 (1.08 to 1.16) |  | 3750 | 2.76 (2.65 to 2.88) |  | 21 730 | 1.08 (1.06 to 1.11) |
| **Vocational students** |  |  |  |  |  |  |  |  |
| F10–F19 Substance use disorders | 678 | 1.14 (1.05 to 1.24) |  | 2370 | 2.98 (2.82 to 3.15) |  | 2704 | 1.08 (1.04 to 1.13) |
| F20–F29 Schizophr. spectr. disorders | 41 | 1.04 (0.76 to 1.42) |  | 594 | 4.65 (4.15 to 5.21) |  | 936 | 1.12 (1.04 to 1.20) |
| F30–F39 Mood disorders | 3447 | 1.07 (1.02 to 1.11) |  | 3804 | 3.21 (3.07 to 3.35) |  | 8103 | 1.05 (1.02 to 1.08) |
| F40–F48 Anxiety disorders | 4870 | 1.07 (1.03 to 1.11) |  | 4188 | 2.57 (2.47 to 2.67) |  | 8717 | 1.07 (1.04 to 1.10) |
| F50 Eating disorders | 98 | 1.13 (0.92 to 1.40) |  | 180 | 2.07 (1.74 to 2.46) |  | 2748 | 1.02 (0.98 to 1.07) |
| F90–F98 Behav. and emot. disorders | 1776 | 1.09 (1.02 to 1.17) |  | 1085 | 2.84 (2.63 to 3.07) |  | 14 308 | 1.08 (1.05 to 1.11) |
| Internalising disorders | 10 792 | 1.08 (1.05 to 1.12) |  | 6026 | 2.88 (2.78 to 2.99) |  | 14 326 | 1.07 (1.04 to 1.10) |
| Externalising disorders | 2553 | 1.10 (1.05 to 1.16) |  | 2675 | 2.98 (2.83 to 3.14) |  | 9135 | 1.07 (1.04 to 1.10) |
| Any of the above | 18 897 | 1.08 (1.05 to 1.11) |  | 7389 | 2.87 (2.78 to 2.97) |  | 20 464 | 1.08 (1.05 to 1.11) |

The models were adjusted for sex, birth year, area-level urbanicity, area-level morbidity, area-level education, area-level employment, number of students in the same ninth-grade class, total number of students in the entire ninth grade, parental education, parental income, parental mental health, and school achievement. For a sensitivity analysis with a restricted time window for mental disorder diagnosis during upper secondary education, see Table S5.

Table S3: Results from the logistic regression analysis with a restricted time window for mental disorder diagnosis during upper secondary education. Associations between having ninth-grade classmates with a mental disorder diagnosis and receiving a mental disorder diagnosis during the first year of upper secondary education (top), and receiving a mental disorder diagnosis during the first year of upper secondary education and dropping out of upper secondary education (bottom).

|  | **Ninth-grade classmates with a mental disorder diagnosis** | | | | | | |  |  |  |
| --- | --- | --- | --- | --- | --- | --- | --- | --- | --- | --- |
|  | **None (ref.)** |  | **One** | |  | **Two** | |  | **Three or more** | |
|  | **n** |  | **n** | **OR (95% CI)** |  | **n** | **OR (95% CI)** |  | **n** | **OR (95% CI)** |
| **Mental disorder diagnosed during upper secondary education** |  |  |  |  |  |  |  |  |  |  |
| Academic students | 1664 |  | 1806 | 1.10 (1.03 to 1.18) |  | 1180 | 1.19 (1.10 to 1.29) |  | 1008 | 1.26 (1.15 to 1.37) |
| Vocational students | 1620 |  | 1819 | 1.13 (1.06 to 1.21) |  | 1196 | 1.20 (1.11 to 1.30) |  | 1099 | 1.27 (1.17 to 1.38) |
|  |  |  |  |  |  |  |  |  |  |  |
|  | **Mental disorder diagnosed during upper secondary education** | | | | | | | | | |
|  | **No (ref.)** |  | **Yes** | |  |  |  |  |  |  |
|  | **n** |  | **n** | **OR (95% CI)** |  |  |  |  |  |  |
| **Dropped out of upper secondary education** |  |  |  |  |  |  |  |  |  |  |
| Academic students | 33 275 |  | 1476 | 3.73 (3.49 to 3.99) |  |  |  |  |  |  |
| Vocational students | 30 176 |  | 1513 | 2.51 (2.36 to 2.68) |  |  |  |  |  |  |

The logistic regression models we adjusted for sex, birth year, area-level urbanicity, area-level morbidity, area-level education, area-level employment, number of students in the same ninth-grade class, total number of students in the entire ninth grade, parental education, parental income, parental mental health, and school achievement.

Table S4: Results from the causal mediation analysis with a restricted time window for mental disorder diagnosis during upper secondary education. Effect of having one or more than one ninth-grade classmate with a mental disorder diagnosis on dropping out of upper secondary education, with one’s own mental disorder diagnosis received during the first year of upper secondary education as a mediator.

|  | **Ninth-grade classmates with a diagnosed mental disorder** | | | | |
| --- | --- | --- | --- | --- | --- |
|  | **One** |  | **Two** |  | **Three or more** |
| **Academic students** |  |  |  |  |  |
| Total effect | 1.06 (1.03 to 1.09) |  | 1.10 (1.06 to 1.15) |  | 1.16 (1.10 to 1.21) |
| Direct effect | 1.06 (1.02 to 1.09) |  | 1.10 (1.06 to 1.14) |  | 1.15 (1.09 to 1.20) |
| Indirect effect | 1.00 (1.00 to 1.00) |  | 1.01 (1.00 to 1.01) |  | 1.01 (1.01 to 1.02) |
| Exposure-mediator interaction | 1.00 (1.00 to 1.01) |  | 1.02 (1.01 to 1.03) |  | 1.02 (1.01 to 1.03) |
| **Vocational students** |  |  |  |  |  |
| Total effect | 1.05 (1.01 to 1.08) |  | 1.11 (1.08 to 1.15) |  | 1.24 (1.18 to 1.30) |
| Direct effect | 1.04 (1.01 to 1.07) |  | 1.10 (1.07 to 1.15) |  | 1.27 (1.17 to 1.30) |
| Indirect effect | 1.00 (1.00 to 1.00) |  | 1.00 (1.00 to 1.01) |  | 1.01 (1.00 to 1.01) |
| Exposure-mediator interaction | 1.00 (0.99 to 1.01) |  | 1.01 (1.00 to 1.02) |  | 1.03 (1.02 to 1.05) |

Odds ratios with 95% CIs for the total effect, (total natural) direct effect, (total natural) indirect effect, and exposure-mediator (reference) interaction shown separately for academic and vocational students. The causal mediation analysis models included the following baseline confounders: sex, birth year, area-level urbanicity, area-level morbidity, area-level education, area-level employment, number of students in the same ninth-grade class, total number of students in the entire ninth grade, parental education, parental income, and parental mental health. School achievement was included as a confounder affected by the exposure.

Table S5: Diagnosis-specific results from the logistic regression analysis with a restricted time window for mental disorder diagnosis during upper secondary education. Associations between (A) having ninth-grade classmates with a mental disorder diagnosis and receiving a mental disorder diagnosis during the first year of upper secondary education, and (B) receiving a mental disorder diagnosis during the first year of upper secondary education and dropping out of upper secondary education.

|  | **A** |  |  | **B** |  |
| --- | --- | --- | --- | --- | --- |
|  | n | OR (95% CI) |  | n | OR (95% CI) |
| **Academic students** |  |  |  |  |  |
| F10–F19 Substance use disorders | 44 | 1.14 (0.84 to 1.56) |  | 119 | 2.88 (2.29 to 3.62) |
| F20–F29 Schizophrenia spectrum disorders | 5 | 1.12 (0.46 to 2.74) |  | 71 | 7.51 (5.26 to 10.71) |
| F30–F39 Mood disorders | 751 | 1.36 (1.24 to 1.48) |  | 757 | 5.18 (4.70 to 5.71) |
| F40–F48 Anxiety disorders | 843 | 1.09 (1.00 to 1.19) |  | 592 | 3.16 (2.85 to 3.49) |
| F50 Eating disorders | 110 | 1.14 (0.93 to 1.39) |  | 137 | 2.86 (2.34 to 3.49) |
| F90–F98 Behavioral and emotional disorders | 462 | 1.09 (0.95 to 1.25) |  | 293 | 4.34 (3.70 to 5.08) |
| Internalising disorders | 2275 | 1.18 (1.11 to 1.26) |  | 1214 | 3.95 (3.67 to 4.26) |
| Externalising disorders | 185 | 1.17 (0.98 to 1.40) |  | 191 | 3.76 (3.10 to 4.56) |
| Any of the above | 3994 | 1.15 (1.09 to 1.23) |  | 1476 | 3.73 (3.49 to 3.99) |
| **Vocational students** |  |  |  |  |  |
| F10–F19 Substance use disorders | 145 | 1.34 (1.12 to 1.59) |  | 319 | 2.16 (1.88 to 2.48) |
| F20–F29 Schizophrenia spectrum disorders | 6 | 1.33 (0.59 to 3.02) |  | 67 | 5.34 (3.80 to 7.49) |
| F30–F39 Mood disorders | 592 | 1.17 (1.06 to 1.29) |  | 574 | 2.97 (2.67 to 3.29) |
| F40–F48 Anxiety disorders | 830 | 1.19 (1.09 to 1.31) |  | 603 | 2.60 (2.35 to 2.87) |
| F50 Eating disorders | 32 | 1.46 (1.00 to 2.12) |  | 38 | 1.82 (1.27 to 2.61) |
| F90–F98 Behavioral and emotional disorders | 580 | 1.06 (0.94 to 1.20) |  | 371 | 2.81 (2.46 to 3.22) |
| Internalising disorders | 2058 | 1.17 (1.10 to 1.25) |  | 1069 | 2.71 (2.51 to 2.93) |
| Externalising disorders | 542 | 1.16 (1.04 to 1.29) |  | 468 | 2.40 (2.13 to 2.70) |
| Any of the above | 4114 | 1.18 (1.11 to 1.25) |  | 1513 | 2.51 (2.36 to 2.68) |

The models were adjusted for sex, birth year, area-level urbanicity, area-level morbidity, area-level education, area-level employment, number of students in the same ninth-grade class, total number of students in the entire ninth grade, parental education, parental income, parental mental health, and school achievement.

Table S6: Diagnosis-specific results from the causal mediation analysis with a restricted time window for mental disorder diagnosis during upper secondary education. Effect of ninth-grade classmates with a mental disorder diagnosis on dropping out of upper secondary education, with one’s own mental disorder diagnosis received during the first year of upper secondary education as a mediator.

|  | **Total effect** | **Direct effect** | **Indirect effect** | **Exposure-mediator interaction** |
| --- | --- | --- | --- | --- |
| **General students** |  |  |  |  |
| F10–F19 Substance use disorders | 1.08 (1.03 to 1.12) | 1.07 (1.03 to 1.12) | 1.00 (1.00 to 1.00) | 1.01 (1.00 to 1.01) |
| F20–F29 Schizophrenia spectrum disorders | 0.99 (0.90 to 1.09) | 0.99 (0.90 to 1.08) | 1.00 (0.99 to 1.00) | 1.00 (0.99 to 1.00) |
| F30–F39 Mood disorders | 1.07 (1.04 to 1.10) | 1.06 (1.03 to 1.09) | 1.01 (1.00 to 1.01) | 1.01 (1.00 to 1.01) |
| F40–F48 Anxiety disorders | 1.05 (1.02 to 1.07) | 1.05 (1.02 to 1.07) | 1.00 (1.00 to 1.00) | 1.01 (1.00 to 1.01) |
| F50 Eating disorders | 0.99 (0.95 to 1.02) | 0.99 (0.95 to 1.02) | 1.00 (1.00 to 1.00) | 1.00 (0.99 to 1.00) |
| F90–F98 Behavioral and emotional disorders | 1.08 (1.05 to 1.10) | 1.07 (1.04 to 1.09) | 1.00 (1.00 to 1.00) | 1.01 (1.00 to 1.01) |
| Internalizing disorders | 1.08 (1.05 to 1.10) | 1.08 (1.05 to 1.10) | 1.01 (1.00 to 1.00) | 1.01 (1.00 to 1.01) |
| Externalizing disorders | 1.09 (1.06 to 1.11) | 1.09 (1.06 to 1.11) | 1.00 (1.00 to 1.00) | 1.01 (1.00 to 1.01) |
| Any of the above | 1.08 (1.05 to 1.09) | 1.07 (1.04 to 1.09) | 1.01 (1.00 to 1.00) | 1.01 (1.00 to 1.01) |
| **Vocational students** |  |  |  |  |
| F10–F19 Substance use disorders | 1.08 (1.03 to 1.13) | 1.08 (1.03 to 1.13) | 1.00 (1.00 to 1.00) | 1.01 (1.00 to 1.01) |
| F20–F29 Schizophrenia spectrum disorders | 1.12 (1.05 to 1.20) | 1.12 (1.05 to 1.20) | 1.00 (0.99 to 1.00) | 1.01 (1.00 to 1.02) |
| F30–F39 Mood disorders | 1.07 (1.04 to 1.10) | 1.07 (1.03 to 1.09) | 1.00 (1.00 to 1.00) | 1.01 (1.00 to 1.01) |
| F40–F48 Anxiety disorders | 1.08 (1.05 to 1.11) | 1.08 (1.05 to 1.11) | 1.00 (1.00 to 1.00) | 1.01 (1.00 to 1.01) |
| F50 Eating disorders | 1.03 (0.98 to 1.08) | 1.03 (0.98 to 1.08) | 1.00 (1.00 to 1.00) | 1.01 (0.99 to 1.01) |
| F90–F98 Behavioral and emotional disorders | 1.09 (1.06 to 1.11) | 1.09 (1.06 to 1.11) | 1.00 (1.00 to 1.00) | 1.01 (1.00 to 1.01) |
| Internalizing disorders | 1.08 (1.05 to 1.10) | 1.08 (1.04 to 1.10) | 1.00 (1.00 to 1.00) | 1.01 (1.00 to 1.01) |
| Externalizing disorders | 1.07 (1.04 to 1.09) | 1.07 (1.03 to 1.09) | 1.00 (1.00 to 1.00) | 1.01 (1.00 to 1.01) |
| Any of the above | 1.09 (1.06 to 1.11) | 1.09 (1.05 to 1.11) | 1.00 (1.00 to 1.00) | 1.01 (1.00 to 1.01) |

Odds ratios with 95% CIs for the total effect, (total natural) direct effect, (total natural) indirect effect, and exposure-mediator (reference) interaction shown separately for academic and vocational students. The causal mediation analysis models included the following baseline confounders: sex, birth year, area-level urbanicity, area-level morbidity, area-level education, area-level employment, number of students in the same ninth-grade class, total number of students in the entire ninth grade, parental education, parental income, and parental mental health. Comprehensive school achievement was included as a confounder affected by the exposure.

## References

1. Ministry of Education and Culture, Finland. (2024, June 14). The Finnish education system. https://okm.fi/en/education-system
